# Myeloid leukemia with t(7;21)(p22;q22) and 5q deletion

**DOI:** 10.3892/or.2013.2623

**Published:** 2013-07-18

**Authors:** IOANNIS PANAGOPOULOS, LUDMILA GORUNOVA, PETTER BRANDAL, MARGARET GARNES, ANNE TIERENS, SVERRE HEIM

**Affiliations:** 1 Section for Cancer Cytogenetics, Institute for Medical Informatics, The Norwegian Radium Hospital, Oslo University Hospital, Oslo, Norway; 2 Centre for Cancer Biomedicine, Faculty of Medicine, University of Oslo, Oslo, Norway; 3 Department of Medicine, Ålesund Hospital, Ålesund, Norway; 4 Department of Pathology, The Norwegian Radium Hospital, Oslo University Hospital, Oslo, Norway; 5 Faculty of Medicine, University of Oslo, Oslo, Norway

**Keywords:** acute myeloid leukemia, cytogenetic, t(7;21)(p22;q22), 5q aberration, *RUNX1*, *USP42*, fusion gene

## Abstract

The rare but recurrent *RUNX1-USP42* fusion gene is the result of a t(7;21)(p22;q22) chromosomal translocation and has been described in 6 cases of acute myeloid leukemia (AML) and one case of refractory anemia with excess of blast. In the present study, we present the molecular genetic analysis and the clinical features of a t(7;21)(p22;q22)-positive AML case. PCR amplified two *RUNX1-USP42* cDNA fragments but no reciprocal *USP42-RUNX1* fragment indicating that the *RUNX1-USP42* is the leukemogenic fusion gene. Sequencing of the two amplified fragments showed that exon 6 or exon 7 of *RUNX1* (accession number NM_001754 version 3) was fused to exon 3 of *USP42* (accession number NM_032172 version 2). The predicted RUNX1-USP42 fusion protein would contain the Runt homology domain (RHD), which is responsible for heterodimerization with CBFB and for DNA binding, and the catalytic UCH (ubiquitin carboxyl terminal hydroxylase) domain of the USP42 protein. The bone marrow cells in the present case also had a 5q deletion, and it was revealed that 5 out of the 8 reported cases (including the present case) with t(7;21)(p22;q22)*/RUNX1-USP42* also had cytogenetic abnormalities of 5q. The fact that t(7;21) and 5q- occur together much more often than chance would allow seems to be unquestionable, although the pathogenetic connection between the two aberrations remains unknown.

## Introduction

It is now generally accepted that neoplastic disorders arise through the acquisition of genomic changes by suitably primed target cells (1). These somatic mutations are often cytogenetically visible in the form of balanced or unbalanced chromosome aberrations (1). Many hematologic malignancies are characterized by balanced chromosomal abnormalities resulting in chimeric genes of pathogenetic, diagnostic and prognostic importance (1). In fact, more than 200 different genes are now known to be rearranged through translocations in leukemias and leukemia-like disorders (1), with some genes being particularly promiscuous, having numerous partners in different fusions and disorders (1).

To date, the *RUNX1* gene (previously *AML1*, *CBFA2* in 21q22) has been shown to fuse in-frame with 23 different partner genes, encoding a structurally heterogeneous group of proteins, in acute myeloid and lymphoblastic leukemia (AML and ALL), chronic myeloid leukemia (CML; the fusion here occurs secondarily), and myelodysplastic syndromes (MDS) (2,3). Some of the fusions are common, such as the *ETV6/RUNX1* [t(12;21)(p13;q22)] in ALL, *RUNX1/RUNX1T1* (also known as *AML1/ETO*) [t(8;21)(q22;q22)] in AML and *RUNX1/MECOM* [t(3;21)(q26;q22)] in MDS, AML and CML in the blastic phase, whereas many of them have only been reported in single cases, i.e., they have not yet been shown to be recurrent (2,3). Whereas the prognostic impact of frequent *RUNX1* fusions is well known, corresponding knowledge regarding infrequent chimeras is lacking (4,5). Considering that most treatment protocols are in part based on the presence of certain genetic changes in acute leukemias, it is of potential clinical value to obtain further information also about rare *RUNX1* fusions, even in disease subgroups that cannot be treated with medications specifically directed against the leukemogenic defect. It is important to underscore that this may be the case also for rare pathogenetic mechanisms where information is gathered by the addition of single case reports, as recently exemplified by the story of the rare *MLL/ARHGAP26 (GRAF)* fusion in pediatric AML (6–8). For this reason, we here present the molecular genetic and clinical features of a case of AML with t(7;21)(p22;q22), a rare but recurrent chromosomal translocation that was first described in 2006 by Paulsson *et al*(9).

## Materials and methods

### Case history

The study was approved by the Regional Committee for Medical Research Ethics (REK Sør, http://helseforskning.etikkom.no), and written informed consent was obtained from the patient.

**Figure 1:**
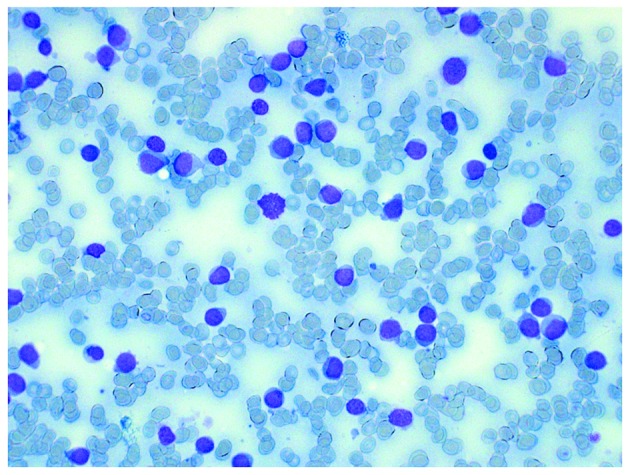
Bone marrow smear of the patient taken at diagnosis. A monomorphous image of blasts which are small and with a scarse cytoplasm is evident. Giemsa staining at magnification ×400.

A 52-year-old woman was admitted to the hospital following a month of tiredness, sleepiness and symptoms of lower airway infection. She had been treated with antibiotics without any clinical improvement. Upon admission to the hospital, she had fever, severe anemia (hemoglobin 5.8 g/l), thrombocytopenia (116×10^9^/l) and leukocytosis (34×10^9^/l). A bone marrow aspirate showed >70% myeloblasts. The immunophenotypical features of the malignant cells confirmed the diagnosis of acute myeloblastic leukemia without differentiation. The myeloblasts expressed CD34, CD117, HLA-DR antigens, CD13, and partly CD11b, in addition to CD7 and CD56, but were negative for myeloperoxidase as well as B- and T-cell lineage markers. The clinical, blood sample and bone marrow findings (Fig. 1) were conclusive for acute myeloblastic leukemia without maturation (formerly FAB M0).

The patient was transferred to the regional hospital and standard induction chemotherapy was administered. Following complete remission five months later, she received an allogenic bone marrow transplant with reduced conditioning (preferred because of complications during initial therapy) from a sibling donor. The patient is, at the time of the preparation of this manuscript, still in remission with a good chimerism status nine months after the primary diagnosis, although she is now being treated for complications due to graft vs. host disease and cytomegalovirus reactivation.

### G-banding and fluorescence in situ hybridization (FISH)

Bone marrow cells were cytogenetically investigated by standard methods. Chromosome preparations were made from metaphase cells of a 24-h culture, G-banded using Leishman’s stain and karyotyped according to ISCN 2009 guidelines (10). As part of our standard cytogenetic diagnosis of AML patients, interphase FISH analyses of bone marrow cells were performed with the Cytocell Multiprobe AML/MDS panel (Cytocell, http://www.cytocell.co.uk/) searching for −5/del(5q), *PML/RARα*, del(17p) (*TP53*), *AML1/ETO*, trisomy 8, −7/del(7q), *CBFβ/MYH11* and del(20q). The del(5q) probe contains the probe for the *EGR1* gene in 5q31.1 labeled in red as well as a control probe at 5p15.31 flanking the marker D5S30 labeled in green. Fluorescent signals were captured and analyzed using the CytoVision system (Applied Imaging, Newcastle, UK).

### PCR analyses

Total RNA (1 *μ*g) was reverse-transcribed in a 20-*μ*l reaction volume using iScript Advanced cDNA Synthesis kit for RT-qPCR according to the manufacturer’s instructions (Bio-Rad). cDNA corresponding to 50 ng total RNA was used as the template in subsequent PCR assays. The 25-*μ*l PCR volume contained 12.5 *μ*l Premix Ex Taq™ DNA Polymerase Hot Start version (Takara), 1 *μ*l of diluted cDNA, and 0.2 *μ*M of each of the forward and reverse primers. For the detection of the *RUNX1-USP42* fusion transcript, the forward RUNX1–765F (GGATGTTCCAGATGGCACTCTGG) and the reverse USP42–562R (ACGTCCCCAGGATTACTGAGTGCC) primers were used. For the amplification of a possible *USP42-RUNX1* fusion transcript, the primers USP42–116F (CAGAAT CAGCCTGGCAGCTCCGA) and RUNX1–1489R (GCCGA CATGCCGATGCCGAT) were used. The PCR was run on a C-1000 Thermal cycler (Bio-Rad) with an initial denaturation at 94°C for 30°sec, followed by 35 cycles of 7°sec at 98°C, 2°min at 68°C, and a final extension for 5°min at 68°C. PCR products (4 *μ*l) were stained with GelRed (Biotium), analyzed by electrophoresis through 1.0% agarose gel and photographed. The remaining PCR products were excised from the gel, purified using the Qiagen Gel extraction kit (Qiagen), and cloned to the pCR4-TOPO vector using TOPO TA cloning kits for sequencing (Invitrogen). Colonies were sequenced at GATC Biotech (Germany, http://www.gatc-biotech.com/en/home.html). The BLAST software (http://www.ncbi.nlm.nih.gov/BLAST/) was used for computer analysis of sequence data.

## Results

**Figure 2:**
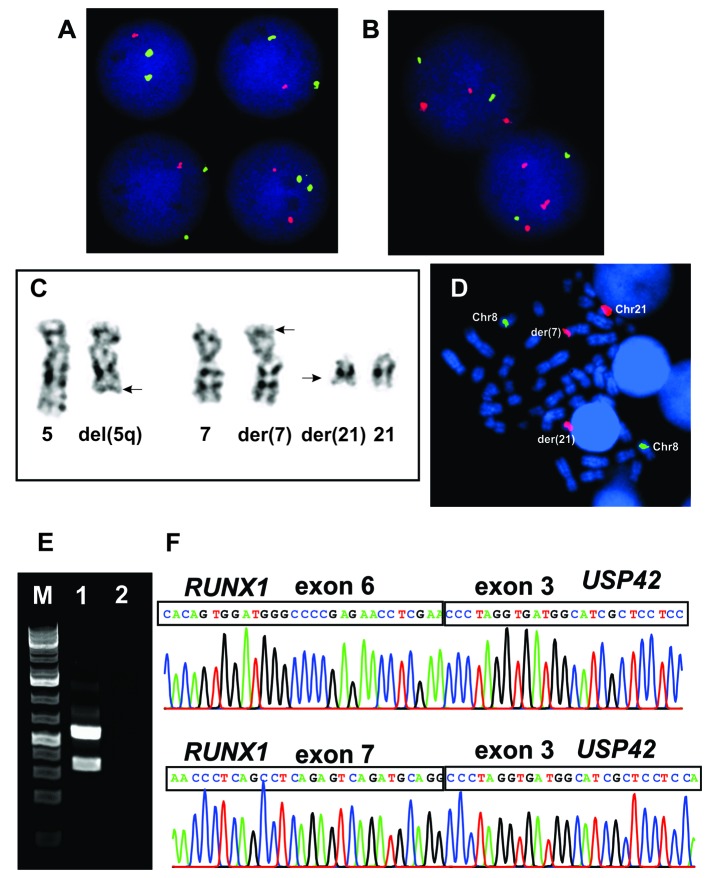
Cytogenetic, FISH and PCR analyses. (A) Interphase FISH with del(5q) probe. The *EGR1* probe (in 5q31) is labeled in red and the control probe mapped in 5p15.31 is labeled in green. Three nuclei had one red signal suggesting a hemizygous deletion of the *EGR1* gene. All four nuclei had two green signals of the control probe. (B) Interphase FISH with the AML1*/*ETO probe. The AML1 probe (*RUNX1*) is labeled in red and the ETO probe (*RUNX1T1*) is labeled in green. Both nuclei had two green signals which suggest that the *RUNX1T1* gene was not rearranged. Both nuclei had three red signals which suggest than one *RUNX1* locus was rearranged. (C) Partial karyotype showing chromosome aberrations del(5q), der(7)t(7;21)(p22;q22), and der(21)t(7;21)(p22;q22) together with the corresponding normal homologues; breakpoint positions are indicated by arrows. (D) FISH on metaphase spread using the AML1/ETO probe. Green signals (ETO probe) are observed only on chromosomes 8 (normal *RUNX1T1*). Part of the AML1 probe (*RUNX1*) is located on 7p22. (E) cDNA fragment amplification of *RUNX1-USP42* (lane 1) using the primers RUNX1–765F and USP42–562R. PCR with primers USP42–116F and RUNX1–1489R did not amplify any cDNA fragment (lane 2). M, 1 kb DNA ladder. (F) Partial sequence chromatograms of the two amplified *RUNX1-USP42* fragments showing that exon 6 of *RUNX1* is fused to exon 3 of *USP42* and that exon 7 of *RUNX1* is fused to exon 3 of *USP42*.

The G-banding analysis showed del(5)(q31) in all 15 cells analyzed which was confirmed by interphase FISH (Fig. 2A and C). The *AML1/ETO* probe (*RUNX1/RUNX1T1*) showed abnormal signals, i.e., splitting of the *RUNX1* probe was observed in the majority of interphase nuclei examined in spite of no cytogenetically visible rearrangement of this chromosome arm (Fig. 2B). In the same experiment two metaphase cells were found which demonstrated that part of the *RUNX1* probe was unexpectedly located on 7p22 (Fig. 2C and D). Other FISH analyses detected no *PML/RARα*, del(17p), trisomy 8, −7/del(7q), *CBFβ/MYH11* or del(20q). Therefore, the whole karyotype was: 46,XX,del(5)(q31)[15].nuc ish (EGR1×1)[196/206],(ETOx2,AML1×3)[186/222].ish t(7;21)(p22;q22) (AML1+;AML1+)[2] (Fig. 1).

PCR amplification using the RUNX1–765F and USP42–562R primers generated two RUNX1-USP42 fragments of 500- and 300-bp in size whereas PCR with primers USP42–116F and RUNX1–1489R did not amplified any cDNA fragment (Fig. 2E). Sequencing of the two amplified fragments showed that, in the 300-bp fragment, exon 6 of *RUNX1* (accession number NM_001754 version 3) was fused to exon 3 of *USP42* (accession number NM_032172 version 2), whereas in the 500-bp long fragment exon 7 of *RUNX1* was fused to exon 3 of *USP42* (Fig. 2F).

## Discussion

The cryptic t(7;21)(p22;q22) chromosomal translocation was first described in a 7-year-old boy with AML-M0 together with aberrant expression of T-lymphocyte-associated markers (9). The translocation was an unexpected finding after FISH had been performed using whole chromosome painting probes for chromosome 7 while screening pediatric leukemias for the t(7;12)(q36;p13) translocation (9). In the present study, we also detected the t(7;21) unexpectedly as a result of our standard cytogenetic diagnosis of AML patients using interphase FISH analyses of bone marrow cells and searching for −5/del(5q), *PML/RARα*, del(17p), *AML1/ETO*, trisomy 8, −7/del(7q), *CBFβ/MYH11* and del(20q). The finding of a split *RUNX1* probe in 186 of 222 interphase nuclei examined triggered further investigations which led to the detection of the t(7;21). Notably, AML with t(7;21) seems to be associated with AML-M0 [our patient was also undifferentiated AML-M0] or myelomonocytic differentiation. One patient was found to present with MDS RAEB-2 (11,12). Since the AML M0 subset makes up <5% of all AMLs (14), it seems likely that the 7;21-translocation is unusual in leading to this particular undifferentiated myeloid leukemia in a dysproportionate number of cases (admittedly, they may also display monocytic differentiation according to a few reports). These leukemias are immunophenotypically characterized by the aberrant expression of CD7 as well as CD56 (present case and 5 previously reported cases) (9,12,13).

Subsequent molecular genetic investigations of the bone marrow cells showed that the result of the t(7;21)(p22;q22) is the fusion of *USP42* (on 7p22) and *RUNX1* (on 21q22) to generate a *RUNX1-USP42* chimera (9). Including the present case, a *RUNX1-USP42* chimera has now been found in 8 cases while the reciprocal *USP42-RUNX1* was noted in 5 (9,11–13). These findings suggest that *RUNX1-USP42* is the leukemogenic fusion. The incidence has hitherto been higher in males (n=6) than in females (n=2) and all but one patient (the first described case) were adults (age >30 years) (9,11–13).

Although recurrent, the *RUNX1-USP42* fusion seems to be rare. Paulsson *et al*(9) screened 35 additional AML cases, Foster *et al*(11) screened 100 AML/MDS with normal karyotypes, and Giguére and Hebert (13) examined 95 leukemias without finding additional cases of *RUNX1-USP42*. Jeandidier *et al*(12) studied 397 AML cases and found only 3 cases with *RUNX1-USP42* fusion. An interesting observation was that all 3 had additional 5q abnormalities resulting in loss of material from that chromosome arm. In total, 6 out of 8 cases (the present one included) with the t(7;21)(p22;q22)*/RUNX1-USP42* fusion had cytogenetically visible changes of 5q resulting in loss of material (9,11–13). In the series presented by Jeandidier *et al*(12), 3 out of 35 leukemias with 5q- had the t(7;21)(p22;q22)/*RUNX1-USP42* fusion gene (8.5%). In only one case was there direct evidence as to whether 5q- or t(7;21) was the primary cytogenetic abnormality. The patient described by Paulsson *et al*(9) had only the t(7;21) at the primary diagnosis (detected as *RUNX1-USP42* fusion), whereas a 5q- occurred secondarily as an additional anomaly in a later sample.

**Figure 3:**
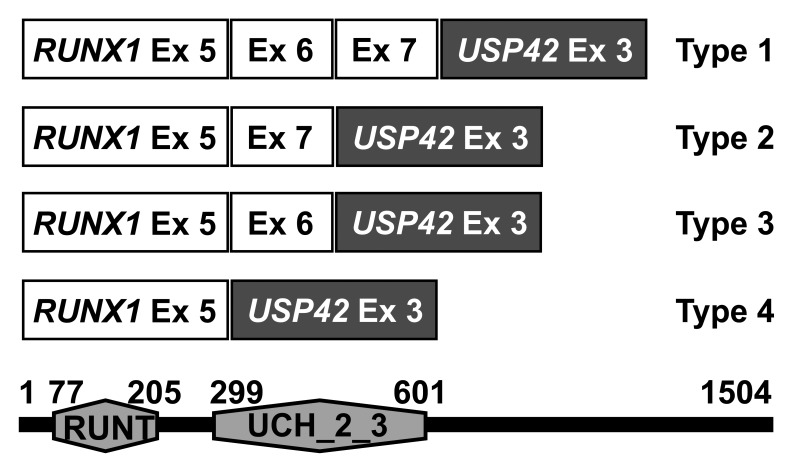
Diagram showing the four known variants of the *RUNX1-USP42* chimeric transcripts and the predicted RUNX1-USP42 protein of 1504 amino acids which corresponds to the type 1 chimeric transcript. The protein retains the RUNT domain of RUNX1 (position 77–205) and the ubiquitin carboxyl-terminal hydrolases family 2 domain (UCH_2_3; position 299–601).

There are 4 types of *RUNX1-USP42* chimeric transcripts, defined here as types 1 to 4 (Fig. 3). The *RUNX1-USP42* type 1 was described as that in which exon 7 of *RUNX1* is fused to exon 3 of *USP42*(9,11). The type 2 was defined as the transcript in which exon 7 of *RUNX1* is fused to exon 3 of *USP42* but exon 6 of *RUNX1* is spliced out from the fusion (9,11). In the type 3 fusion transcript, exon 6 of *RUNX1* is fused to exon 3 of *USP42*. In type 4, finally, exon 5 of *RUNX1* is fused to exon 3 of *USP42*(11). In all *RUNX1-USP42* fusion transcripts, the predicted fusion protein would be expected to contain the Runt homology domain (RHD) which is responsible for heterodimerization with CBFB and DNA binding, and the catalytic UCH (ubiquitin carboxyl terminal hydroxylase) domain of the USP42 protein (9,11). The function of this fusion protein and its cellular consequences leading to leukemia are unknown. It might exert its leukemogenic effect as other RUNX1 fusions do in which the RHD is retained but the transactivation domain of RUNX1 is removed, i.e., by acting as a dominant-negative inhibitor of wild-type RUNX1 in transcription activation (3). In fact, these RUNX1 fusions mimic the RUNX1α variant which has higher affinity to DNA binding but suppresses the transcription activation of RUNX1β (3). RUNX1-USP42 might also affect the regulation of TP53 since the USP42 interacts and deubiqitinates this protein (15). More information concerning the cellular function of the normal USP46 is clearly needed in order to understand the role of *RUNX1-USP42* fusions in leukemias.
